# Supplementary material for: Content-rich biological network constructed by mining PubMed abstracts
Source: BMC Bioinformatics. 2004 Oct 8;5:147. doi: 10.1186/1471-2105-5-147 (PMC528731; doi:10.1186/1471-2105-5-147)
Supplement: Additional File 5 — The original Chilibot query results of the term "long-term potentiation (LTP)" and 22 other terms, limiting the latest references analyzed to the years 1990, 1995, 2000, and 2004. [file 1471-2105-5-147-S5.bz2 › chilibotAdditionalFile5/ltp2004/html/SYNAPSIN I.html]

 


**SYNAPSIN I** (Input: SYNAPSIN I ) 

---


**Google Searches:** Entire Web  | EDU domain only  | PDF files only

.

**External Links:** OMIM |
LocusLink
| Swissprot
| GeneCards

  
**Maps of SYNAPSIN I**

Simple
Complete
graph in
radiant
tree
square
layout.

**New Hypothesis !**

**Synonyms** 

- synapsin i   [PubMed]

**Synopsis**

- These results suggest significant contribution of **synapsin I** to the formation and maintenance of the presynaptic structure.  J Cell Biol, 1995 
  [23]

- In particular, animals maintained on the HFS diet showed a decrease in levels of **synapsin I** mRNA and protein, important for neurotransmitter release.  Neuroscience, 2002 
  [23]

- These results suggest that glutamate can activate CaM kinase II through NMDA receptors in the induction of LTP and in turn stimulates the phosphorylation of target proteins such as MAP2 and **synapsin I**.  Nippon Yakurigaku Zasshi, 1993 
  [20]

- These results suggest that the increased level of **synapsin I** mRNA is related to persistent enhancement of synaptic activity within the neural networks in which dentate granule cells participate in LTP.  Brain Res, 1998 
  [20]

- We have previously shown that the induction of long term potentiation  [LTP]  LTP at perforant path to dentate granule cell synapses in the rat hippocampus induces changes in the mRNA levels of syntaxin 1B and **synapsin I**, known to be involved in neurotransmitter release.  Eur J Neurosci, 1998 
  [20]

- SynapsinI and synapsin II are widely expressed synaptic vesicle phosphoproteins that have been proposed to play an important role in synaptic transmission and synaptic plasticity.  Neuropharmacology, 1995 
  [19]

- In cultured hippocampal neurons, these proteins are present in dendrites and are often found near the presynaptic protein, **synapsin I**.  Proc Natl Acad Sci U S A, 2002 
  [19]

- The selectivity of the changes in trkB and **synapsin I**, affecting the hippocampus, is in agreement with the role of this structure in processing of spatial information.  Brain Res, 2001 
  [19]

- Quantitative immunohistochemical analysis revealed that **synapsin I** increased significantly in the molecular layer of the dentate gyrus and the stratum lucidum of CA3, suggesting activation of the granule cells by synaptic input to the dentate gyrus, and persistent enhancement of glutamate release from the axon terminals of the dentate granule cells.  Brain Res, 2000 
  [16]

- Exercise reversed the decrease in BDNF and its downstream effectors on plasticity such as **synapsin I**, a molecule with a key role in the modulation of neurotransmitter release by BDNF, and the transcription factor cyclic AMP response element binding protein CREB, important for learning and memory.  Neuroscience, 2004 
  [16]

- Studies of knockout mice have shown that the synaptic vesicle protein Rab3A is required for mossy fibre LTP, but the protein kinase A substrates rabphilin, **synapsin I** and synapsin II are dispensable.  Nature, 2002 
  [16]

- We hypothesize that the beta adrenergic agonist stimulated phosphorylation of **synapsin I** and synapsin II in young rats plays a role in the increase in transmitter release produced by NE in the dentate.  Proc Natl Acad Sci U S A, 1991 
  [16]

- Our results indicate that LTP expression is accompanied by persistent changes in presynaptic phosphorylation, and specifically that presynaptic CaM kinase II activity and **synapsin I** phosphorylation may be involved in LTP expression.  Proc Natl Acad Sci U S A, 1996 
  [11]

- In fact, BDNF potentiates intracellular calcium release presynaptically and BDNF induced TrkB activation results in phosphorylation of **synapsin I** via mitogen activated protein kinase, which increases the number of synaptic vesicles available for release.  Sheng Li Xue Bao, 2003 
  [11]

- These results support the idea that CaM kinase II contributes to the induction of hippocampal LTP in both postsynaptic and presynaptic regions through phosphorylation of target proteins such as the AMPA receptor, MAP2 and **synapsin I**.  Neurochem Int, 1996 
  [11]
